# Transcriptome Profiling of A549 Xenografts of Nonsmall-cell Lung Cancer Treated with Qing-Re-Huo-Xue Formula

**DOI:** 10.1155/2022/2882801

**Published:** 2022-09-16

**Authors:** Zexi Lv, Xiqun Chen, Kai Yang, Yuhang Zhao, Jie Cui, Wuniqiemu Tulake

**Affiliations:** ^1^Department of Integrative Medicine, Huashan Hospital, Fudan University, Shanghai, China; ^2^Institute of Integrative Medicine, Fudan University, Shanghai, China

## Abstract

Lung cancer is one of the most common malignant tumors, and non-small cell lung cancer (NSCLC) accounts for 85% of all lung cancer cases. Chinese herbal formula Qing-Re-Huo-Xue (QRHXF) has shown antitumor effects in the NSCLC xenograft mouse model of Lewis cells. However, the molecular mechanisms underlying the antitumor effects of QRHXF remain unknown. In this study, an A549 xenograft mouse model was established, and the mice were then treated with QRHXF or vehicle through oral gavage. Tumor growth was monitored. Tumors were subsequently harvested, and RNA sequencing was performed. Compared with the control group, mice treated with QRHXF showed smaller tumor size and slower tumor growth. RNA sequencing results indicated 36 differentially expressed genes between QRHXF treated and control groups. 16 upregulated and 20 downregulated genes were identified. Enrichment analysis showed four differential expression genes (DEGs) related to tumor growth pathways RASAL2, SerpinB5, UTG1A4, and PDE3A. In conclusion, this study revealed that QRHXF could inhibit tumor growth in an A549 xenograft mouse model, and the target genes of QRHXF may include PDE3A, RASAL2, SERPIB5, and UTG1A4.

## 1. Introduction

Lung cancer is one of the most frequent malignancies and the leading cause of cancer-related deaths worldwide. There are 20 million new cases of lung cancer and 17.9 million deaths each year [1]. Non-small cell lung cancer (NSCLC) accounts for about 85% of all lung cancer cases [2]. Along with the advances in our understanding of disease biology, the discoveries of new predictive biomarkers have altered outcomes for many NSCLC patients [3]. For early-stage NSCLC patients, the main treatment is a combination of surgery and chemotherapy [2]. For patients with advanced NSCLC, the standard treatment is combined treatment with radiotherapy and chemotherapy [4]. NSCLC patients undergoing chemotherapy and radiotherapy display poor quality of life and poor prognosis [5]. Although the treatment of NSCLC improved in the past 25 years, the prognosis is still unsatisfactory [6]. The predicted five-year survival rate for NSCLC is still low (23%) compared to breast cancer (89.6%) and prostate cancer (98.2%) [7]. Thus, the development of more efficient treatment is needed.

Traditional Chinese medicine (TCM) has been used to treat various diseases [8], and it has shown effects in the treatment of lung cancer [9]. Many Chinese herbs and herb extracts have been shown to have antitumor effects. For example, Gambogenic acid extracted from Chinese herb gamboge was demonstrated to induce cell death in NSCLC cells [10]. Baicalin extracted from Radix Scutellariae, and paeoniflorin extracted from Radix Paeoniae were reported to have antitumor effects in lung cancer [11–13]. QRHXF is an empirical prescription developed by our institute. It is derived from “Treatise on Febrile and Miscellaneous Diseases.” The formula consists of two Chinese herbs Radix Paeoniae (RP, chi-Shao in Chinese), the dried root of Paeonia *lactiflora* Pall, and Radix Scutellariae (RS, Huang-qin in Chinese), the dried root of Scutellaria baicalensis. Our team has reported tumor-inhibitory effects of QRHXF using a C57BL/6 mouse xenograft model [14], QRHXF suppressed cancer progression by inhibiting the tumor-associated macrophages. The present study reports the transcriptomic profile of A549 xenografts of NSCLC treated with QRHXF.

## 2. Materials and Methods

### 2.1. Animals

Mice used in this research were 6-week-old male BALB/c-nu inbred nude mice. Mice were kept in pathogen-free cages with a temperature of 26–28°C, and the relative humidity of the room was kept at 40–60%. Mice were housed at four per cage with 12-hour light/dark cycles.

### 2.2. Reagents and Drugs

QRHXF granules were purchased from Jiangyin Tianjiang Pharmaceutical Co. (Jiangsu province, China). Fetal bovine serum, trypsin, culture medium, and RNAlater™ Stabilization Solution (AM7021), Quantitative real-time PCR kits (4367659) were purchased from Thermo Fisher Scientific.

### 2.3. Cell Culture

A549 human NSCLC cells were cultured in Dulbecco's minimum essential medium (D-MEM) with 10% fetal bovine serum and 100 U/ml penicillin-streptomycin. The culture was maintained at 37°C with 5% CO_2_.

### 2.4. NSCLC Xenograft Mouse Model

A549 cells were resuspended and inoculated into the armpit of mice. About three weeks later, the tumors were removed and cut into small pieces (< 1 mm^3^). For each mouse, one piece of tumor tissue was implanted into the armpit of a mouse with a trocar. The mice were divided into four groups control group, QRHXF low-dose group (2.5 g/kg), QRHXF medium-dose group (5 g/kg), and QRHXF high-dose group (10 g/kg). Each group contained nine mice. The control group was given saline once daily by gavage, the experimental groups were given corresponding doses of QRHXF once daily by gavage. Body weight and tumor volume were monitored every 3–4 days.

### 2.5. Sample Processing

The mice were sacrificed at 33 days following the treatment by cervical dislocation, and the tumors were dissected immediately. Tumors from three randomly selected mice from each group for RNA seq analysis. The tissues were cut into 5 mm pieces and soaked in RNAlater, and then frozen at −20°C after 4°C overnight.

TaKaRa MiniBEST Universal RNA Extraction Kit was used to extract total RNA from tumor tissues, and then the RNA concentration was assessed with NanoDrop (Thermo Fisher Scientific). TaKaRa PrimeScript™ RT Master Mix was used in RNA reverse transcription. The reaction condition was as follows: 37°C for 15 minutes, 85°C for 5 seconds. The RNA concentration was measured by NanoDrop2000 (Thermo Fisher Scientific). Gel electrophoresis was used to analyze the integrity of total RNA, the 28 s/18 s value should be more than 1.5, and the RNA integrity number (RIN) should be more than 7. RNA sequencing was performed by Shanghai Biotechnology Corporation.

### 2.6. Differential Expression Analysis

Differential expression analysis between the control group and the QRHXF medium-dose group was conducted. Analysis of differential expression genes (DEGs) was repeated twice. The false discovery rate (FDR) was used to determine *p* value threshold. The q value is the corrected *p* value. Log2 was used to transform FPKM values, the results were shown as fold change.

### 2.7. Functional Analysis

Kyoto Encyclopedia of Genes and Genomes (KEGG) analysis was performed using the KEGG pathway database (https://www.genome.jp/kegg/pathway.html), the number of DEGs in each pathway was counted, and pathway enrichment analysis was conducted.

### 2.8. Quantitative Real-Time PCR

The reaction condition was set as the instruction of power SYBR Green Master Mix (ABI, 4367659). Primers used in the study were as follows: RASAL2-F: AGCAGAAAGGTCCCCTCGTAG, RASAL2-R: AGGGTGAGGTATTTGCAGTGT, SerpinB5-F: AATTCGGCTTTTGCCGTTGAT, SerpinB5-R: TGTCACCTTTAGCACCCACTT, UGT1A4-F: TTTGTCTTCCAATTACATGC, UGT1A4-R: AGATATGGAAGCACTTGTAAG, PDE3A-F: CCACGGCCTCATTACCGAC, PDE3A-R: TTGCTCACGGCTCTCAAGG.

### 2.9. Statistical Analysis

The results were expressed as mean ± SD. Tumor volume differences between groups were evaluated by two-way ANOVA. Between-group differences in body weight and tumor weight were accessed by one-way ANOVA. Statistics analyses were performed using GraphPad Prism software (version 7.0). A *p* value < 0.05 was considered significant.

### 2.10. Western Blot Analysis

The total protein was normalized by BCA Protein Assay Kit (P0012, Beyotime Biotechnology, Shanghai, China) and then separated by SDS-PAGE. The samples were incubated with corresponding antibodies for 1 h and detected with an enhanced chemiluminescence substrate (Tanon, Shanghai, China). Antibodies: ACTIN (3700T, Cell signaling technology, USA), PDE3A (YN0061, Immunoway, Suzhou, China).

## 3. Results

### 3.1. QRHXF Inhibited Tumor Growth In Vivo

**Figure 1:**
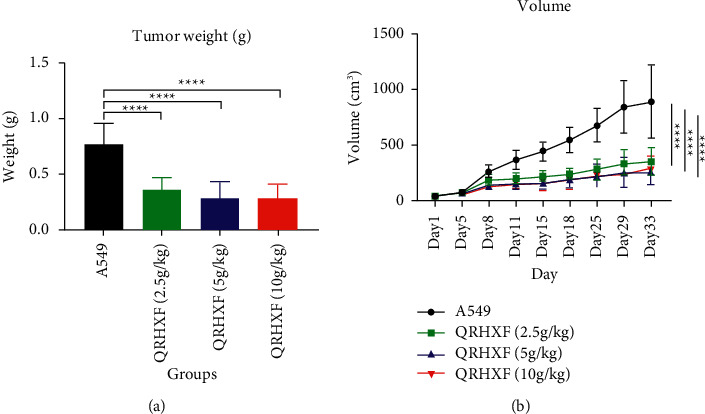
Tumor weight and tumor volume. Nude mice were used to establish the A549 xenograft tumor mouse model, the control group was given saline by gavage, and the experimental groups were given different doses of QRHXF. Each mouse was administered once a day for 33 days. Tumor weight (a) and tumor volume (b) in different groups. ^*∗∗∗∗*^*p* < 0.0001.

To identify the antitumor effect of QRHXF, we established a NSCLC xenograft model with A549 cells using 6-week-old BALB/c-nu mice. The tumor weights were compared between different groups. As shown in Figure 1, compared with the control group, tumor weight decreased in all groups treated with QRHXF. But there was no significant difference between different doses of QRHXF. These results suggested that QRHXF could inhibit tumor growth in the A549 xenograft model.

### 3.2. Differentially Expressed Genes between QRHXF Medium-Dose Group and Control Group

**Figure 2:**
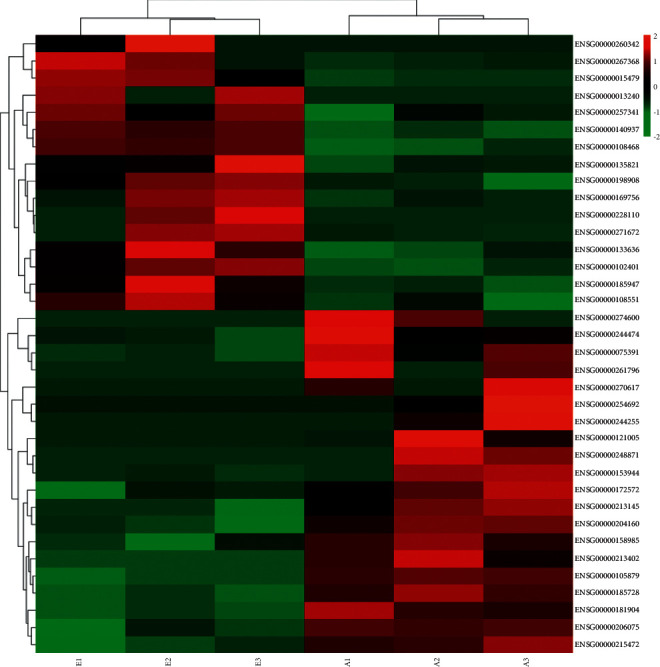
Clustering heat map of differential expression genes in each sample. The A549 xenograft tumor mouse model was performed as previously described. Three tumor samples from the control group and Qing-Re-Huo-Xue formula medium-dose group were randomly selected for RNA sequencing. In the heatmap, red represents upregulated differential genes, green represents downregulated differential genes, and black represents no significant difference genes.

**Figure 3:**
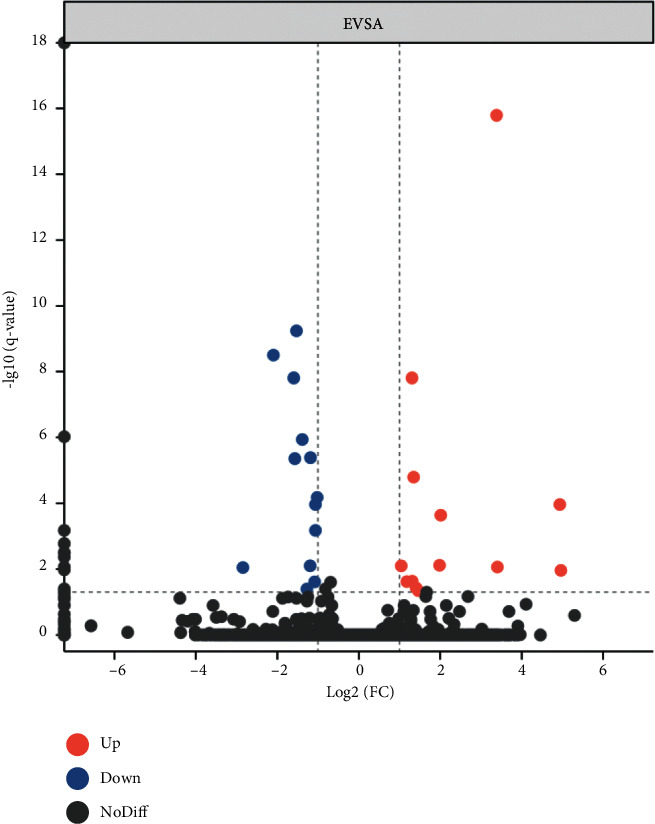
Volcano plot of differential expression genes. According to the results of RNA sequencing, we obtained 36 differential expression genes in total with a *p* value <  0.05 and Log2(FC) > 2, of which 16 were upregulated while 20 were downregulated. Red represents upregulated differential genes, green represents downregulated differential genes, and black represents no significant difference genes.

Three samples from the medium-dose group and three samples from the control group were randomly selected to conduct the RNA sequencing. Gene expression differences between the QRHXF medium-dose group and control group (E VS A) were shown in Figure 2. Thirty-six DEGs were identified, of which 16 genes were upregulated and 20 were downregulated. For example, RASAL2 (RAS protein activator like 2), SerpinB5 (mammary serine protease inhibitor B5), UTG1A4 (UDP-glucuronosyltransferase 1A4), PDE3A (phosphodiesterase 3A). The list of DEGs was reported in Table S1. The q values (corrected *p* value) were ≤ 0.05. The statistical significance and fold change were shown in the volcano plot in Figure 3. Upregulated genes were labeled red, while downregulated one was labeled blue. The screening conditions of DEGs were Log_2_ (FC) ≥ 2; q value (corrected *p* value) ≤ 0.05.

### 3.3. KEGG Enrichment Analysis for the Differentially Expressed Genes

**Figure 4:**
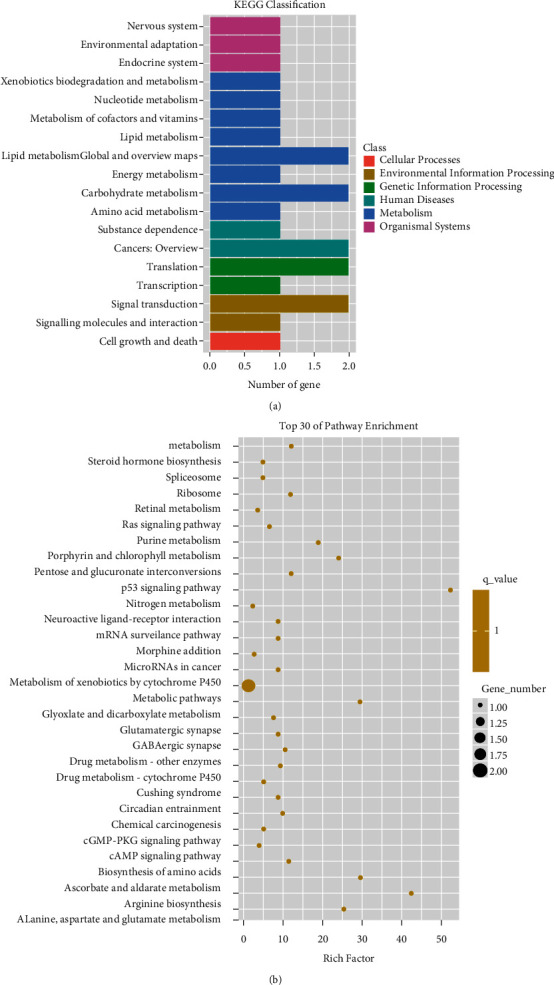
KEGG enrichment analysis of differential expression genes. (a) The KEGG classification. (b) The top 30 high-enrichment KEGG pathways.

KEGG enrichment analyses were performed, and the results were shown in Figure 4. DEGs were enriched in 32 KEGG pathways. The top 30 pathway enrichment were listed in Figure 4. Several tumor-related pathways were enriched. Such as chemical carcinogenesis, the cAMP signaling pathway, microRNAs in cancer, and the p53 signaling pathway.

### 3.4. Verification of Selected DEGs

**Figure 5:**
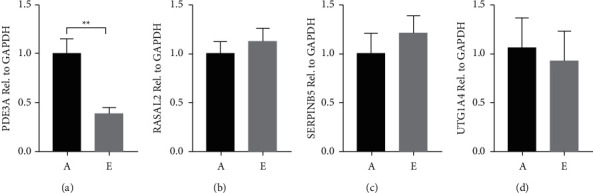
qPCR validation results of four selected genes: PDE3A (a) RASAL2 (b) SerpinB5 (c) and UTG1A4 (d). ^*∗∗*^*p* < 0.01.

To verify the results of RNA sequencing, four genes related to the tumor were further assessed by qPCR RASAL2 from Ras signaling pathway, UGT1A4 from chemical carcinogenesis, PDE3A from cAMP signaling pathway, SerpinB5 from microRNAs in cancer, and p53 signaling pathway. These pathways play important roles in cell proliferation. As shown in Figure 5 mRNA level of PDE3A was reduced in QRHXF medium-dose group compared with the control group. The expression changes of PDE3A were consistent with the sequencing results (Figure S1). The qPCR verification results of the other three genes were inconsistent with the sequencing results. The inconsistent verification results of RASAL2, UGT1A4, and SerpinB5 may be due to the small sample size.

## 4. Discussion

In this study, we found that the A549 xenograft mouse model treated with QRHXF exhibited a smaller tumor size and less tumor weight compared with the control group.

QRHXF is consist of two Chinese herbs *Radix Paeoniae* and *Radix Scutellariae*. 6′-O-galloylpaeoniflorin, a bioactive compound extracted from the roots of *Radix Paeoniae*, was reported to regulate the miR-299-5p/ATF2 axis and inhibit the proliferation of A549 cells [4]. Baicalin, baicalein, and wogonin are three major flavonoids extracted from *Radix Scutellariae* [5,6]. Baicalin was reported to enhance the antitumor activity of factor-related apoptosis-inducing ligands via p38 activation and ROS accumulation [7]. It also inhibited H-460 cell proliferation, inhibited tumor growth, and promoted survival in an H-460 xenograft mouse model [8]. Additionally, baicalein exerted antitumor function through the Src/Id1 pathway in an A549 orthotopic lung cancer model [9]. Wogonin was reported to act as a cisplatin sensitizer for cancer therapy [10]. The major isoflavone of *Radix Paeoniae* and *Radix Scutellariae* has been shown to have antitumor effects. A previous study reported that QRHXF suppressed tumor progression by inhibiting tumor-associated macrophages [11]. We used A549 cells and nude mice to establish a xenograft mouse model and observed slower growth of the xenografts in QRHXF-treated mice, providing additional evidence that QRHXF has potential as an antitumor formula.

We employed RNA sequencing to explore the downstream pathways of the QRHXF action. The results indicated that there were 36 DEGs. Four genes (RASAL2, SERPINB5, UTG1A4, and PDE3A) were selected for validation using quantitative qPCR. These 4 genes were screened from enriched tumor-related pathways. PDE3A was significantly downregulated, consistent with the sequencing result. However, RASAL2, SERPINB5, and UTG1A4 showed nonsignificant changes compared with the control group. The discrepancy might be explained by the difference in methodologies that RNA sequencing may be more sensitive in detecting transcript changes.

PDE3A is a member of the cGMP-inhibited cyclic nucleotide phosphodiesterase family. Studies indicated that PDE3A mediated tumor suppressive effect of anagrelide [12]. Breast tumor cells were found sensitive to PDE3A inhibitors [13], and expression of PDE3A was negatively correlated with breast cancer prognosis. The interaction between PDE3A and schlafen 12 protein could induce apoptosis in Hela cells and Hela xenografts tumors [14]. In chemoresistant A549 cells, PDE3A was significantly downregulated, and high PDE3A expression was associated with favorable overall survival and progression-free survival in adenocarcinoma patients [15]. We found that the tumor-inhibitory effects of QRHXF were accompanied by PDE3A downregulation, and this result was validated by qPCR, indicating a possible role of PDE3A in the QRHXF action.

RASAL2 is a member of the Ras GTPase-activating protein family and negatively regulates the RAS signaling pathway [16]. It promotes colorectal cancer progression via the hippo pathway [17], and the knockdown of RASAL2 inhibits the growth and invasion of hepatocellular carcinoma cells [18]. In addition, activation of the RASAL2/ARHGAP24/RAC1 module slows triple-negative breast cancer (TNBC) progression [19], and RASAL2 overexpression is related to poor prognosis and tumor recurrence in TNBC. However, previous studies also suggested RASAL2 as a tumor suppressor [20]. We found decreased RASAL2 expression in A549 xenograft tumors treated with QRHXF due to the RNA sequencing results. SerpinB5, also known as maspin [21] is a member of the serine protease inhibitor superfamily [22]. It is involved in many biological processes, including cell adhesion, apoptosis [23], protein degradation, oxidative stress [24], and embryonic development [25]. SerpinB5 has been reported to inhibit cell motility, invasion, and metastasis in breast cancer cell models [26,27]. In NSCLC, maspin seemed to be similarly controversial. Some studies suggest strong maspin expression as a favorable factor in NSCLC [28,29]. The exact role of serpinB5 is unclear. UGT1A4 is one of the subfamilies of UDP-glucuronosyltransferase enzymes (UGTs), which catalyze the phase II metabolic pathway [30]. It is currently known that UTG1A members can conjugate drugs or carcinogens [31,32]. The exact functional roles of RASAL2, SERPINB5, UTG1A4, and PDE3A in NSCLS and in the antitumor effects of QRHXF require further investigations.

## 5. Conclusion

Our study revealed that QRHXF could inhibit tumor growth, and this effect of QRHXF may involve PDE3A, RASAL2, SERPIB5, and UTG1A4. Further characterization of the specific functional roles of the 4 genes and other altered genes is necessary to better understand the molecular mechanisms underlying the beneficial impact of QRHXF on NSCLC. This study provided a theoretical basis for the clinical treatment of NSCLC with the Qing-Re-Huo-Xue formula, and also provided a basis for the study of the role of PDE3A in NSCLC.

## Data Availability

The data used to support the findings of this study are included within the article.

## Supplementary Materials

Supplementary Materials Table S1. 36 enriched up- and downregulated DEGs. Figure S1. Western blotting validation of PDE3A. The protein levels of PDE3A in the NSCLC samples were determined by Western blot assays. Representative Western blot images with ACTIN as a loading control are shown.
