# Supplementary material for: Transcriptome Profiling of A549 Xenografts of Nonsmall-cell Lung Cancer Treated with Qing-Re-Huo-Xue Formula
Source: Evid Based Complement Alternat Med. 2022 Sep 16;2022:2882801. doi: 10.1155/2022/2882801 (PMC9507736; doi:10.1155/2022/2882801)
Supplement: Supplementary Materials — Table S1. 36 enriched up- and downregulated DEGs. Figure S1. Western blotting validation of PDE3A. The protein levels of PDE3A in the NSCLC samples were determined by Western blot assays. Representative Western blot images with ACTIN as a loading control are shown. [file 2882801.f1.zip › table s1.pdf]

Gene id	Gene name	Qvalue		updown
ENSG00000140937	CDH11	1.61E-16		UP
ENSG00000185728	YTHDF3	5.73E-10		DOWN
ENSG00000181904	C5orf24	3.13E-09		DOWN
ENSG00000105879	CBLL1	1.54E-08		DOWN
ENSG00000108468	CBX1	1.54E-08		UP
ENSG00000213402	PTPRCAP	9.50E-07		DOWN
ENSG00000215472	RPL17-C18orf32	1.15E-06		DOWN
ENSG00000204160	ZDHHC18	4.09E-06		DOWN
ENSG00000158985	CDC42SE2	4.38E-06		DOWN
ENSG00000102401	ARMCX3	1.59E-05		UP
ENSG00000206075	SERPINB5	6.61E-05		DOWN
ENSG00000261796	ISY1-RAB43	0.000109		DOWN
ENSG00000267368	UPK3BL	0.000109		UP
ENSG00000257341	AL928654.7	0.00023		UP
ENSG00000213145	CRIP1	0.000669		DOWN
ENSG00000274600	RIMBP3B	0.000669		DOWN
ENSG00000270617	URGCP-MRPS24	0.001681		DOWN
ENSG00000254692	RP11-468E2.1	0.003124		DOWN
ENSG00000260342	RP11-1035H13.3	0.003124		UP
ENSG00000213240	RP11-458D21.5	0.004283		UP
ENSG00000135821	GLUL	0.007592		UP
ENSG00000172572	PDE3A	0.007892		DOWN
ENSG00000015479	MATR3	0.00793		UP
ENSG00000133636	NTS	0.008595		UP
ENSG00000248871	TNFSF12-TNFSF13	0.008595		DOWN
ENSG00000244474	UGT1A4	0.00889		DOWN
ENSG00000228110	ST13P19	0.010471		UP
ENSG00000271672	DUXAP8	0.010868		UP
ENSG00000108551	RASD1	0.02314		UP
ENSG00000185947	ZNF267	0.02388		UP
ENSG00000075391	RASAL2	0.024323		DOWN
ENSG00000169756	LIMS1	0.037182		UP
ENSG00000121005	CRISPLD1	0.039823		DOWN
ENSG00000153944	MSI2	0.040155		DOWN
ENSG00000198908	BHLHB9	0.045102		UP
ENSG00000244255	XXbac-BPG116M5.17	0.046028		DOWN
