# Supplementary figures and images for: Transcriptome Profiling of A549 Xenografts of Nonsmall-cell Lung Cancer Treated with Qing-Re-Huo-Xue Formula
Source: Evid Based Complement Alternat Med. 2022 Sep 16;2022:2882801. doi: 10.1155/2022/2882801 (PMC9507736; doi:10.1155/2022/2882801)

**Group A**

**Group E**

**ACTIN**

**PDE3A**

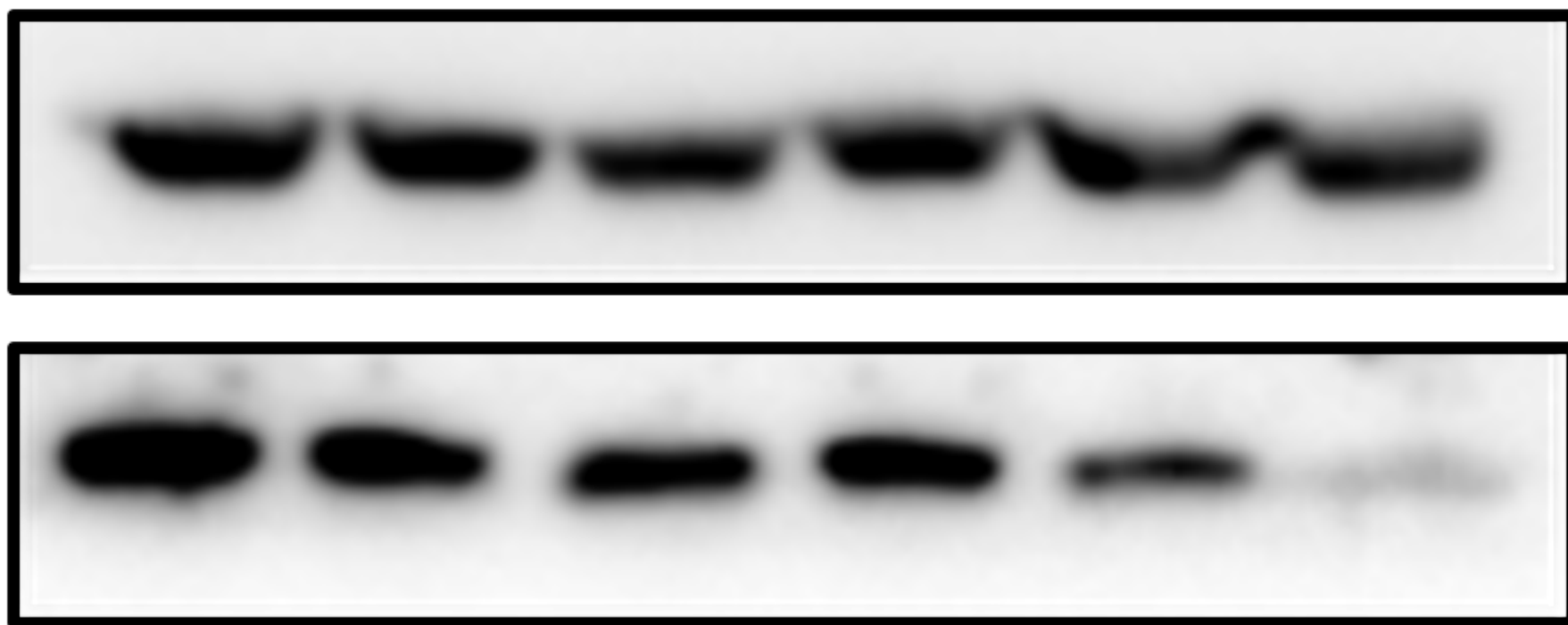

Supplement: Supplementary Materials — Table S1. 36 enriched up- and downregulated DEGs. Figure S1. Western blotting validation of PDE3A. The protein levels of PDE3A in the NSCLC samples were determined by Western blot assays. Representative Western blot images with ACTIN as a loading control are shown. [file 2882801.f1.zip › figure s1.pdf]
